# Effects of Different β-Lactam Antibiotics on Indirect Tomato (*Solanum lycopersicum* L.) Shoot Organogenesis and *Agrobacterium tumefaciens* Growth Inhibition In Vitro

**DOI:** 10.3390/antibiotics10060660

**Published:** 2021-06-01

**Authors:** Nataliya V. Varlamova, Yuliya I. Dolgikh, Andrey O. Blinkov, Ekaterina N. Baranova, Marat R. Khaliluev

**Affiliations:** 1 Laboratory of Plant Cell Engineering, All-Russia Research Institute of Agricultural Biotechnology, Timiryazevskaya 42, 127550 Moscow, Russia; nv_varlamova@rambler.ru (N.V.V.); yulia53@list.ru (Y.I.D.); aoblinkov@gmail.com (A.O.B.); 2 Laboratory of Cell Biology, All-Russia Research Institute of Agricultural Biotechnology, Timiryazevskaya 42, 127550 Moscow, Russia; greenpro2007@rambler.ru; 3 Laboratory of Plant Protection, N.V. Tsitsin Main Botanical Garden of Russian Academy of Sciences, Botanicheskaya 4, 127276 Moscow, Russia; 4 Department of Biotechnology, Institute of Agrobiotechnology, Russian State Agrarian University—Moscow Timiryazev Agricultural Academy, Timiryazevskaya 49, 127550 Moscow, Russia

**Keywords:** *Solanum lycopersicum* L., Claforan^®^, timentin, amoxicillin, Amoxiclav^®^, clavulanic acid, callus induction, shoot regeneration, agar disk-diffusion method

## Abstract

A β-lactams that act by inhibiting the bacterial cell wall biosynthesis are one of the most common classes of antibiotics applied to suppress the growth of latent bacterial infection associated with the plant tissue culture, as well as in the *Agrobacterium*-mediated transformation techniques. Plant sensitivity to antibiotics usually is species-, genotype-, or even tissue-specific and mainly depends on concentrations, growth conditions, and culture system. In the presented article, we estimated a comparative effect of four β-lactam antibiotics (Claforan^®^, timentin, amoxicillin, and Amoxiclav^®^) at different concentrations in an agar-solidified Murashige and Skoog (MS) culture medium supplemented with 5 mg L^−1^ 6-benzylaminopurine (6-BA) and 0.1 mg L^−1^ indole-3-acetic acid (IAA) on in vitro callus induction and shoot organogenesis from hypocotyl and cotyledon explants of two tomato cultivars (Rekordsmen, Moryana). The role of clavulanic acid in combination with amoxicillin (Amoxiclav^®^) in the shoot organogenesis frequency and number of shoots per explant has been demonstrated. Additionally, the growth inhibition of *Agrobacterium tumefaciens* AGL0 strain according to agar disk-diffusion assay was studied. As a result, both stimulatory (timentin, amoxicillin, and Amoxiclav^®^) and inhibitory (Claforan^®^) effects of β-lactam antibiotics on in vitro morphogenetic responses of tomato were noted. It was found that clavulanic acid, which is part of the commercial antibiotic Amoxiclav^®^, significantly increased the shoot regeneration frequency from cotyledon and hypocotyl explants of Rekordsmen tomato cultivar. Possible reasons for the stimulating effect of clavulanic acid on the induction of shoot organogenesis are discussed. According to agar disk-diffusion assay, the maximum diameter of growth inhibition zones (43.9 mm) was identified using 200 mg L^−1^ timentin. The in vitro antibacterial activity of tested β-lactam antibiotics was arranged in the following order: timentin > Claforan^®^ > amoxicillin ≥ Amoxiclav^®^. Thus, to suppress the growth of internal and latent bacterial infection of tomato plant tissue culture, as well as for transformation of Moryana and Rekordsmen cultivars by *A. tumefaciens* strain AGL0, we recommend adding of 100–200 mg L^−1^ timentin or 400–800 mg L^−1^ Amoxiclav^®^ to the shoot induction medium.

## 1. Introduction

*Agrobacterium*-mediated transformation is the commonly used method for stable integration of foreign genes into the genome and consequent generations of tomato (*Solanum lycopersicum* L.) and many other dicotyledonous transgenic plants [1,2]. Direct methods to introduce of foreign DNA into the tomato genome, such as bioballistic transformation [3,4], microinjection-based transformation [5], electroporation-mediated transformation [6], and PEG-mediated protoplast transformation [7,8], are used much less frequently. This is due to the fact that *Agrobacterium*-mediated transformation has a number of significant advantages compared with direct methods of introducing exogenous DNA. These include the relative simplicity of transformation procedure and lower cost of equipment, high competence of tomato somatic cells for *Agrobacterium* infection, the ability to transfer a large DNA fragments with low-copy number transgene integration into the plant genome, and stable Mendelian inheritance [2,9].

Regardless of the genetic transformation techniques, the regeneration of full-fledged and fertile shoots from various tomato explants in vitro is a prerequisite. Most frequently, shoot regeneration is achieved from tomato somatic cells via indirect organogenesis through the callus phase [1,10,11,12]. Other approaches for shoot regeneration in vitro in tomato through direct organogenesis [13,14] as well as direct or indirect somatic embryogenesis [12,15,16,17] are much less common.

Plant morphogenesis is a complicated and integrated process, a regulation which is carried out at the cellular, tissue, organ, and organismal level. The in vitro morphogenetic capacity of various somatic tomato tissues is genetically controlled [18,19,20,21]. The morphogenetic responses of tomato somatic cells during *Agrobacterium*-mediated transformation highly depends on the genotype, physiological age and explant type, culture medium as well as quantitative and qualitative component of plant growth regulators (PGRs), *Agrobacterium* strain, type of plasmid vector, and different physical and cultural conditions, as well as other factors [1,22,23].

*A. tumefaciens* is a gram-negative soil bacterium that is pathogenic to plant tissue cultures [2]. Therefore, *Agrobacterium*-mediated transformation can undoubtedly be considered a combination of biotic (contamination of pathogenic bacteria) and abiotic (explant wounding, prolonged cultivation on culture medium supplemented with PGRs and antibiotics at high concentrations for callus induction, shoot regeneration, as well as selection of transgenic plants) stress factors that has a complex multiple effect [24]. Stressful conditions initiate primary stress-induced response, displayed in reactive oxygen species generation and activation of signaling cascades, which ultimately significantly reduces the regeneration and transformation capacity [25]. It was previously demonstrated that the nanomolar concentrations of antioxidant SkQ1, which penetrates the cell membrane and accumulates in mitochondria, stimulated indirect shoot organogenesis of *Zea mays* L., *Triticum aestivum* L., *Saccharum officinarum* L., and *Medicago* sp. [26]. 

Co-cultivation of tomato explants with *Agrobacterium* is carried out mainly at a low temperature (15–18 °C) in darkness for 48–72 h. It is believed that during this time period, the entire cycle of *Agrobacterium*-mediated transformation occurs, from T-DNA excision to insertion into the plant genome. An increase in the co-cultivation period (96 h or more) and temperature leads to intensive multiplication of bacterial cells, premature contamination, and death of explants [27,28]. Tissue browning and necrotization of explants inoculated with *Agrobacterium* in the early stages of cultivation are due to plant cells’ death caused by the plant–pathogen interaction [29]. In this regard, subculturing explants after the co-cultivation stage on a culture medium supplemented with antibiotics is essential for suppressing *Agrobacterium* growth. 

β-lactams (penicillins, cephalosporins, carbapenems, monobactams, and β-lactamase inhibitors) that act by inhibiting the bacterial cell wall biosynthesis [30,31] are one of the most common classes of antibiotics applied to suppress the growth of internal and latent bacterial infection associated with the plant tissue culture, as well as in the *Agrobacterium*-mediated transformation techniques. Clavulanic acid derived from *Streptomyces clavuligerus* is a β-lactam molecule that has little intrinsic antibacterial activity that functions as a mechanism-based β-lactamase inhibitor. Potassium clavulanate (clavulanic acid as a salt of potassium) combined with β-lactam antibiotics such as amoxycillin (trade names Augmentin^®^, Amoxiclav^®^, Clavamox^®^, and others) or ticarcillin (trade name Timentin^®^) to fight antibiotics resistance by preventing their degradation by β-lactamase enzymes, broadening their spectrum of susceptible to bacterial infections [32]. The choice of antibiotic is based on the following requirements: (1) affordable price, (2) highly bacteriostatic and bactericidal effects, (3) no negative effect on the growth and morphogenesis of plant tissue cultures, (4) stability in liquid or solidified culture medium; (5) stability in stock solutions after storage at −20 or −80 °C and periodic thaw–freeze cycles [33]. 

The presented article is devoted to the comparative effect of various β-lactam antibiotics, such as cefotaxime, amoxycillin, and ticarcillin, on in vitro callus induction and shoot organogenesis of two tomato cultivars (Rekordsmen, Moryana), as well as growth inhibition of the *Agrobacterium tumefaciens* AGL0 strain. In addition, the role of clavulanic acid in the form of a potassium salt on tomato morphogenesis was evaluated. 

## 2. Results

### 2.1. The Effects of Different β-Lactam Antibiotics on In Vitro Tomato Morphogenesis

#### 2.1.1. Callus Induction and Frequency of Its Formation

**Figure 1:**
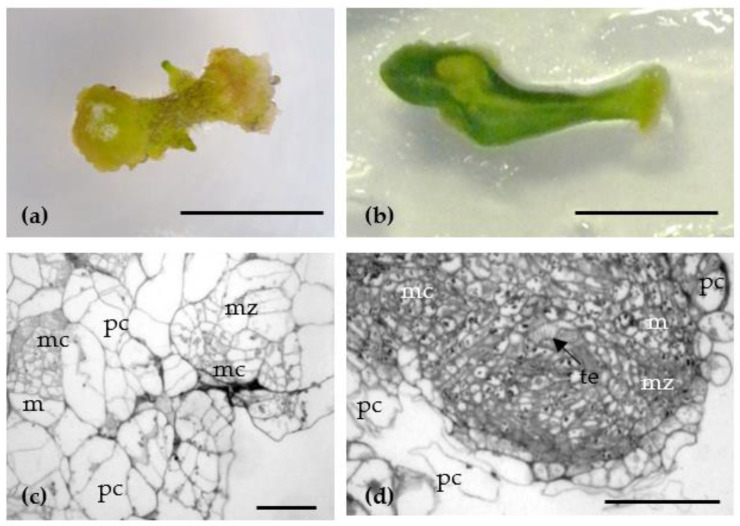
Callus formation from hypocotyl (**a**) and cotyledon (**b**) explants on MS1 culture media supplemented with 5 mg L^−1^ 6-benzylaminopurine (6-BA) and 0.1 mg L^−1^ indole-3-acetic acid (IAA) and its histological analysis (**c**,**d**). Abbreviations: mz—meristematic zone; mc—meristematic cell; pc—parenchyma cell; te—tracheal element. Bars—1 cm (**a**,**b**) and 50 μm (**c**,**d**).

Cotyledon and hypocotyl explants of both tomato cultivars increased in size at the beginning of passaging when cultivated on Murashige and Skoog medium (MS) with PGRs (control MS1) and experimental culture media supplemented with a β-lactam antibiotic (MS2–MS9) (Table 1). Thickening of the overgrown tissues on explants could be observed already on the fifth day of cultivation, from which the callus was subsequently formed. Dense and light green calli were formed on the 10–12 days after planting on the abaxial part of cotyledons, as well as on the edge of cotyledon petioles. The formation of callus tissue on tomato hypocotyl occurred on the entire explant surface. The histological analysis of callus tissue revealed formation of a meristematic zone among hypertrophied cortical parenchyma cells. The meristematic zones consist of meristematic cell masses as well as de novo formed tracheal elements located in their central part (Figure 1). 

**Figure 2:**
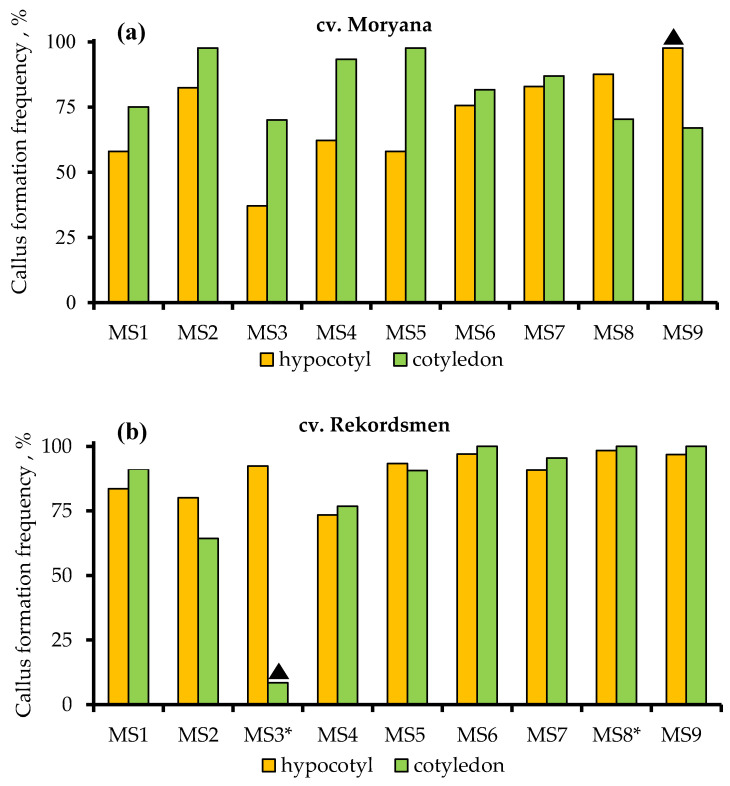
Influence of MS culture media supplemented with different types and concentrations of β-lactam antibiotics (MS2–MS9) on the callus formation frequency from hypocotyl and cotyledon explants of tomato cv. Moryana (**a**) and Rekordsmen (**b**). Abbreviations: ▲—variants of culture media that have significant differences (α = 0.05) between cotyledon and hypocotyl explants; *—variants of culture media significantly different from the control treatment (MS1).

The results of the three-way ANOVA test showed statistical differences at 5% significance level in callus formation frequency between different tomato explants and the studied culture media. In addition, the differences were significant for the interaction of factors “culture medium × genotype”, “culture medium × explant”, “genotype × explant”, and “culture medium × explant × genotype” (Table S1). The frequency of callus formation from hypocotyl and cotyledon explants of Moryana and Rekordsmen cultivars on control MS1 culture medium was 58.0% and 75.0%, as well as 83.6% and 91.3%, respectively (Figure 2). 

Significant differences in the frequency of callus formation between hypocotyl (97.6%) and cotyledon (67.0%) explants in Moryana cultivar were revealed on the culture medium (MS9) supplemented with 800 mg L^−1^ of Amoxiclav^®^. In contrast, a dramatic inhibition of callus formation (8.5%) from cotyledons of Rekordsmen tomato cultivar was observed during the cultivation on a MS3 medium supplemented with a high concentration of cefotaxime. No significant differences were found between the culture media on the average callus formation frequency for two explant types of Moryana tomato cultivar. Similar data were noted for the Rekordsmen cultivar, except for MS3 and MS8 culture media, on which the values of callus formation frequency were, respectively, significantly lower and higher in comparison with the control.

#### 2.1.2. Efficiency of Tomato Shoot Organogenesis 

The efficiency of tomato shoot organogenesis was assessed by the frequency of somatic organogenesis (Table 2), as well as the average number of regenerated shoots per explant (Table 3). The frequency of tomato shoot organogenesis from hypocotyl and cotyledon explants of Moryana and Rekordsmen cultivars on control MS1 culture medium supplemented with PGRs was 4.4% and 1.5%, as well as 67.1% and 42.7%, respectively (Table 2). When explants of the Moryana cultivar were cultured on most media with the addition of antibiotics, no significant differences were found in the shoot organogenesis frequency both in comparison with control and among themselves according to Duncan’s multiple range test. The exception was MS4 and MS6 culture media supplemented with 100 mg L^−1^ timentin and 200 mg L^−1^ amoxicillin, respectively, which stimulated the shoots organogenesis from hypocotyl explants, increasing their frequency by 8.5 and 6.8 times. In contrast to the MS7 culture medium containing 400 mg L^−1^ amoxicillin, the MS8 medium, characterized by the presence of clavulanic acid in the commercial antibiotic Amoxiclav^®^, significantly increased the shoot regeneration frequency from cotyledon and hypocotyl explants of Rekordsmen tomato cultivar. 

A more pronounced effect of β-lactam antibiotics on the induction of somatic shoot organogenesis from different tomato explants was demonstrated for Rekordsmen cultivar. Adding timentin (MS4, MS5), Amoxiclav^®^ (MS8, MS9), and amoxicillin at a concentration of 200 mg L^−1^ (MS6) to the culture medium leads to a significant increase in the shoot organogenesis frequency of from hypocotyl explants (17.6–77.8%). A similar response was observed for the cotyledon explants of Rekordsmen cultivar in the MS5, MS8 and MS9 culture medium. At the same time, the frequency of somatic shoot organogenesis from cotyledon explants on a culture medium containing Amoxiclav^®^ (MS8, MS9) was 100% compared with control treatment (42.7%), and one significantly exceeded other experimental variants. Cultivation of cotyledon explants on MS2 medium supplemented with 400 mg L^−1^ Claforan^®^ dramatically reduced rates of shoot organogenesis (1.2%), while a twofold increase in its concentration completely inhibited the regenerative process.

Both stimulatory and inhibitory effects of β-lactam antibiotics were noted when evaluating the average number of regenerated shoots per explant (Table 3). Compared to the control, a significant increase in the number of regenerated shoots per hypocotyl explant (2.89–3.24) was found on MS5, MS6, and MS8 culture media only for the Rekodsmen cultivar. For cotyledon explants of both tomato cultivars, the stimulatory effect was established for most variants of culture media. At the same time, the average number of shoots produced per cotyledon explant for Moryana and Rekodsmen cultivars varied from 3.2 and 5.6 to 5.6 and 9.2, respectively. 

Thus, on average for both explant types and tomato genotypes, the addition of timentin, amoxicillin, and Amoxiclav^®^ to t MS1 culture medium increased the shoot organogenesis frequency, as well as the number of shoots produced per explant. It was found that cotyledons of both tomato genotypes were characterized by a higher frequency of shoot organogenesis (74.2% and 56.4% for Moryana and Rekodsmen cultivars, respectively) and average shoot number produced per explant (4.1 and 3.5 for Moryana and Rekodsmen cultivars, respectively) than hypocotyl segments. Thus, it is not found significant differences between tomato genotypes on regenerative capacity for both explant types and variants of culture media. 

### 2.2. The Effects of Different β-Lactam Antibiotics on A. tumefaciens Growth Inhibition In Vitro

**Figure 3:**
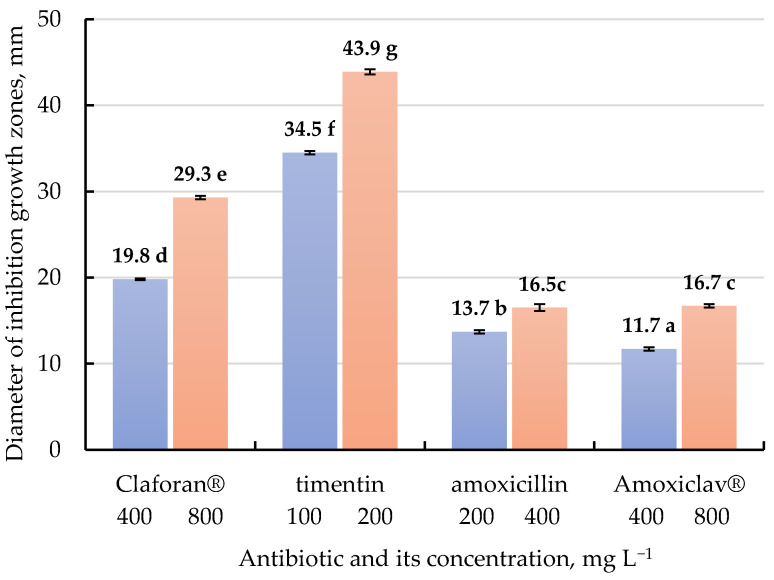
Influence of different β-lactam antibiotics on *A. tumefaciens* growth inhibition (strain AGL0) using agar disk-diffusion assay. The figure shows mean values ± standard errors. Means followed by the same letter are not significantly different at α = 0.05 according to the Duncan’s multiple range test.

The results shown in Figure 3 and Figure S1 represent the antibacterial activity of different β-lactam antibiotics in solidified Luria-Bertani (LB) medium against growth inhibition of *A. tumefaciens* strain AGL0 using agar disk-diffusion assay. Depending on the concentration and type of antibiotic, the diameter of growth inhibition zones varied from 11.7 to 43.9 mm. The higher antibiotic concentration in LB medium increased diameter of the growth inhibition zones. Thus, the antibacterial activity of tested β-lactam antibiotics was arranged in the following order: timentin > Claforan^®^ > amoxicillin ≥ Amoxiclav^®^. 

## 3. Discussion

Optimization of an effective protocol for tomato regeneration system in vitro as well as *Agrobacterium*-mediated transformation is still routine, since morphogenesis efficiency is largely dependent on genetic, physiological, and physical factors [1,5,10,11,12,13,14,15,16,17,21,22,23]. To suppress bacterial infection during the introduction of plant material into an in vitro culture, clonal micropropagation and *Agrobacterium*-mediated transformation plant material is long-term subcultured on a medium supplemented with antibiotics. This is because the cultivation of explants on nutritionally-rich media stimulates an increase of microorganism titer in the cytoplasm, intercellular spaces, and vascular tissues, which ultimately leads to the visible manifestation after n-number of passages, necrosis, and death of plant tissue. *Agrobacterium* contamination of plant tissues does not allow the detection of the true transgenic status of transformants by molecular genetic techniques. In addition, endophytic bacterial microorganisms associated with the plant tissue culture (latent bacterial contamination) determine the regeneration and transformation capacity [34,35]. 

The comparative effect of various types and concentrations of β-lactam antibiotics to inhibit the *Agrobacterium* growth in vitro [36,37] and *in planta* [38,39,40,41,42], as well as on tomato morphogenesis responses [43,44,45], has been previously shown. In pioneering works devoted to the introduction of foreign DNA into the tomato genome by *Agrobacterium*-mediated transformation, the authors used classical carbenicillin [46,47]. With the advent of next generation semi-synthetic β-lactams, such as cefotaxime (Claforan^®^) [22,48,49], cefoxitin (mefoxin) [39], meropenem and imipenem [38,39], moxalactam [39], amoxicillin (Augmentin^®^, Amoxiclav^®^, Clavamox^®^) [42,50], and ticarcillin (Timentin^®^) [10,41,51], singly or coupled with β-lactamase inhibitor (clavulanic acid), they have been utilized at different concentrations to eliminate bacteria during tomato transformation. A number of studies have demonstrated the effective combined use of two overgrowth-control antibiotics in tomato regeneration medium, e.g., 250 mg L^−1^ cefotaxime and 500 mg L^−1^ carbenicillin [23], as well as 250 mg L^−1^ cefotaxime and ticarcillin [40]. In addition to antimicrobial activity, antibiotics have a pronounced positive effect on the regeneration capacity in tomato tissue culture [40,41,42,43,44,45] and many other crops [52,53,54,55,56]. However, their role in stimulating the morphogenesis in vitro is not fully understood, but it has been assumed that the antibiotics or degradation products mimic PGRs, since some of them possess an auxin-like structure [52,56,57].

In our earlier experiments, we evaluated different PGRs on callus induction and shoot organogenesis in vitro from hypocotyl and cotyledon explants of Rekordsmen cultivar and other commercial tomato genotypes [10,11]. In order to reveal the possible stimulating and inhibiting effects of various β-lactam antibiotics on tomato morphogenesis, in this study, we used the MS culture medium supplemented with 5 mg L^−1^ 6-BA and 0.1 mg L^−1^ IAA (MS1), which does not provide high efficiency of shoot regeneration. As a result, culture media were identified that negatively (MS3 medium supplemented with 800 mg L^−1^ of cefotaxime) or positively (MS8 supplemented with 400 mg L^−1^ of Amoxiclav^®^) influenced the callus formation of Rekordsmen cultivar. Several studies have demonstrated no obvious inhibition effect of cefotaxime on callus induction and growth in tomato [41], but it significantly reduced shoot regeneration [41,42,45,49,50]. Kazemi et al. [49] suggested that reducing the negative effects of cefotaxime on tomato regeneration frequency and shoot number per cotyledonary explant was detected when replacing FeEDTA (iron chelate) with FeEDDHA (iron(3^+^)[ethylenediamine-N,N′-bis(hydroxyphenylacetic acid)]) in MS culture medium. In contrast, a stimulatory effect of cefotaxime in maize callus cultures [53] and carrot protoplast cultures [56] has been previously demonstrated. In our case, the addition of Claforan^®^ at different concentrations in the MS1 culture medium dramatically reduced shoot organogenesis or completely inhibited the regenerative process. 

Furthermore, our study identified different concentrations of β-lactams, such as timentin, amoxicillin, and Amoxiclav^®^, that significantly increased the shoot organogenesis frequency, as well as the number of shoots produced per explant from tomato tissue culture. It was found that clavulanic acid, which is part of the commercial antibiotic Amoxiclav^®^, significantly increased the shoot regeneration frequency from cotyledon and hypocotyl explants of Rekordsmen tomato cultivar. A stimulatory effect of ticarcillin and amoxicillin in tomato tissue cultures has also been previously shown [41,42,43,44,45,50]. Since ticarcillin is metabolized to phenylacetic acid, a naturally occurring weak auxin [57], increasing timentin concentration might improve tomato regeneration potential. Clavulanic acid is rapidly hydrolyzed at a 25 °C via the reactive amino ketone (1-amino-2-oxo-butan-4-ol) to pyrazine end-products (2,5-bis(2-hydroxyethyl)pyrazine, 3-carboxyethyl-2,5-bis(2-hydroxyethyl)pyrazine, and 3-ethyl-2,5-bis(2-hydroxyethyl)pyrazine) as well as carbon dioxide and acetaldehyde [58,59]. The stimulating effect of clavulanic acid on morphogenetic responses of tomato is most likely associated with direct action or products of its degradation as catalysts alone or combined with other β-lactam antibiotics or PGRs. Enhanced regenerative capacity may be due to stimulation of these antibiotics on the number of meristematic zones formed in callus tissue, from which subsequent shoot organogenesis occurred. Thus, the obtained results agree with the previously presented data that plant sensitivity to antibiotics usually is species-, genotype-, or even tissue-specific and mainly depends on concentrations, growth conditions and culture system. 

Agar disk-diffusion assay is the official routine in vitro method used in many clinical trials and laboratory testing for antimicrobial susceptibility evaluation of antibiotics and other protective compounds [60,61,62,63]. With this assessment method, we evaluated the effectiveness of four β-lactam antibiotics on the growth elimination of *A. tumefaciens* strain AGL0 at concentrations that are most often applied in tomato transformation. As a result, the maximum diameter of growth inhibition zones was identified using 200 mg L^−1^ timentin (43.9 mm). The in vitro antibacterial activity of tested β-lactam antibiotics was arranged in the following order: timentin > Claforan^®^ > amoxicillin ≥ Amoxiclav^®^. On the basis of agar disk-diffusion assay, the high efficiency of timentin at minimal concentrations (50 mg L^−1^) in inhibiting the growth of *A. tumefaciens* strain EHA105 compared with carbenicillin and cefotaxime was previously demonstrated [37]. Determination of the minimum inhibitory concentration (MIC) and minimum bactericidal concentration (MBC) revealed a high in vitro antibacterial activity of meroperem (carbapenem antibiotic) against *A. tumefaciens* strains AGL0, LBA4404, C58, and EHA101 [36,38]. Ogawa and Mii [36] found that the antibacterial activities of 12 β-lactams tested against strain LBA4404 were equal to or higher than those tested against strain EHA101. The mentioned above indicates the need for screening the type and concentration of antibiotic depending on the *Agrobacterium* strain, taking into account its negatively effects on the morphogenesis of plant tissue cultures.

## 4. Materials and Methods

### 4.1. Plant Material

Two commercial tomato (*S. lycopersicum* L.) cultivars (Rekordsmen and Moryana) were used in the experiment for obtaining donor seedlings. Tomato seeds were kindly provided by the All-Russian Research Institute of Irrigated Vegetable, Melon, and Ground Growing (Astrakhan oblast, Kamyziyak, Russia).

### 4.2. Explant Source, Antibiotics, and Culture Conditions

The seeds were surface sterilized in 96% ethanol for 30 s and in 20% solution (*v*/*v*) of the commercial bleach ‘Ace’ with a few drops of Tween-20 for 7 min, then rinsed with sterilized distilled water five times for 1 min each. After surface sterilization, the seeds were cultured on MS basal medium [64] without PGRs supplemented with 3% (*w*/*v*) sucrose (PanReac AppliChem, Madrid, Spain) and 0.7% (*w*/*v*) agar (PhytoTechnology Laboratories, Lenexa, KS, USA). The pH was adjusted with 1M KOH solution to 5.7–5.8 before autoclaving. The culture medium was sterilized by autoclaving at 121 °C and 1.1 atm for 20 min. 

Whole cotyledons with 2–3 mm petioles as well as hypocotyl segments (approximately 10–15 mm in length) excised from the middle part of 10–12-day-old aseptic seedlings were used as explants. For callus induction and shoot regeneration, the explants were cultivated on agar-solidified MS medium supplemented with 5 mg L^−1^ 6-benzylaminopurine (6-BA) (Sigma-Aldrich, St. Louis, MI, USA) and 0.1 mg L^−1^ indole-3-acetic acid (IAA) (Sigma-Aldrich, St. Louis, MI, USA) (MS1) as well as on MS1 culture medium containing four types of β-lactam antibiotics at different concentrations (MS2–MS9) (Table 1). 

Antibiotics and PGRs were dissolved in distilled water, filter-sterilized (0.22 μm Millipore, Burlington, MA, USA), and stored until use at −20 °C. They were added after autoclaving to a culture medium cooled to 45 °C. Hypocotyl segments and cotyledons were placed in the Petri dishes horizontally and with the abaxial surface in contact with the culture medium, respectively. The explants were subcultured to fresh medium every 15 days. Donor seedlings and explants were kept in a climatic test chamber (Sanyo WLR-351H, Osaka, Japan) at 25 ± 2 °C under a 16-h photoperiod and light intensity of 65 μmol m^−2^ s^−1^.

### 4.3. Histological Analysis of Callus Tissue

Fragments of callus tissue formed from hypocotyl and cotyledon explants on MS1 culture media supplemented with 5 mg L^−1^ 6-BA and 0.1 mg L^−1^ IAA were fixed for 24 h in 2.5% glutaraldehyde (Merck, Darmstadt, Germany) dissolved in 0.1 M Sorensen’s phosphate buffer (pH 7.2) with 1.5% sucrose. Then, the samples were washed, post-fixed in 1% OsO4 (Sigma-Aldrich, USA), and dehydrated in ethanol of increasing concentrations (30%, 50%, 70%, 96%, and 100%) and in propylene oxide (Fluka, Hamburg, Germany). The samples were embedded in mixture of Epon-812 and Araldite (Merck, Germany) according to the standard procedure. For light microscopy, semi-thin sections (1–2 μm) were prepared using glass knives and ultramicrotome LKB-V (LKB, Sweden), placed on glass slides, and embedded in epoxide resin. Samples were photographed using Olympus BX51 microscope (Olympus, Tokyo, Japan) with Color View II camera (Soft Imaging System, Münster, Germany).

### 4.4. Agar Disk-Diffusion Method

The agar disk-diffusion method was used to determine the *Agrobacterium* growth inhibition in vitro by various antibiotics at different concentrations. *A. tumefaciens* strain AGL0 was grown in a 250-mL capacity conical glass flask overnight at 28 °C in 50 mL of LB liquid medium [65] containing 50 mg L^−1^ rifampicin in a rotary shaker (150 rpm). An overnight culture of AGL0 strain was resuspended to OD_600_ = 0.6 and subsequently diluted 1000 times with LB liquid medium. Then, a diluted *Agrobacterium* suspension was inoculated on the surface of glass Petri dishes (100 × 15 mm) containing 25 mL agar-solidified LB medium supplemented with 50 mg/L rifampicin. To the sterile filter paper discs (10 mm in diameter), 50 μL solutions of different antibiotic concentrations such as Claforan^®^, timentin, amoxicillin, and Amoxiclav^®^ (Table 1) were added. A sterile disc without antibiotic was used as negative control. Subsequently, filter paper discs were placed on the surface of agar-solidified LB medium. The Petri dishes were incubated at 37 °C for 48 h, then diameters of inhibition growth zones were measured. 

### 4.5. Accounting Data and Statistical Analysis

To assess the effect of antibiotics on tomato morphogenesis, the frequency of callus formation and shoot organogenesis as well as the average number of shoots per explant were determined using following formulas:

Callus formation frequency (%) = [number of explants successfully forming callus/total number of explants] × 100;

Shoot organogenesis frequency (%) = [number of regenerating explants/number of explants forming callus] × 100;

Average number of shoots per explant = [shoot number/number of regenerating explants] × 100.

Evaluation of the morphogenetic potential was carried out on the 45th day of cultivation. Each variant of treatment was performed in three replications. For one replication, 10 cotyledons and 15 hypocotyl segments per each variant of culture medium were used. Experimental data were assessed at 5% significance level using the analysis of variance (ANOVA) and Duncan’s multiple range tests with AGROS software (version 2.11, Russia). Data of callus formation and shoot organogenesis (%) were arcsin $\sqrt{X}$ transformed prior to statistical analysis. The average number of shoots per explant were $\sqrt{X+1}$ transformed prior ANOVA [66]. 

To assess the effect of varying antibiotic concentrations for eliminating *Agrobacterium*, the diameters of inhibition growth zones were measured. The agar disk-diffusion test was performed three times in three biological replicates (three Petri dishes with three filter paper discs for each variant). 

## 5. Conclusions

In the presented article, we estimated a comparative effect of various β-lactam antibiotics, such as cefotaxime, amoxycillin, and ticarcillin at different concentrations, on in vitro callus induction and shoot organogenesis from hypocotyl and cotyledon explants of two tomato cultivars (Rekordsmen, Moryana). Additionally, the growth inhibition of *A. tumefaciens* AGL0 strain according to agar disk-diffusion assay was implemented. As a result, both stimulatory (timentin, amoxicillin, and Amoxiclav^®^) and inhibitory (Claforan^®^) effects of β-lactam antibiotics on in vitro morphogenetic responses of tomato were noted. It was found that clavulanic acid, which is part of the commercial antibiotic Amoxiclav^®^, significantly increased the shoot regeneration frequency from cotyledon and hypocotyl explants of Rekordsmen tomato cultivar. The stimulating effect of clavulanic acid on morphogenetic responses of tomato is most likely associated with direct action or products of its degradation as catalysts alone or combined with other β-lactam antibiotics or PGRs. Thus, the obtained results agree with the previously presented data that plant sensitivity to antibiotics usually is genotype- and tissue-specific and mainly depends on its concentrations. According to agar disk-diffusion assay, the maximum diameter of growth inhibition zones (43.9 mm) was identified using 200 mg L^−1^ timentin. The in vitro antibacterial activity of tested β-lactam antibiotics was arranged in the following order: timentin > Claforan^®^ > amoxicillin ≥ Amoxiclav^®^. Thus, to suppress the growth of internal and latent bacterial infection associated with tomato plant tissue culture, as well as for tomato transformation of Moryana and Rekordsmen cultivars by *A. tumefaciens* strain AGL0, we recommend adding 100–200 mg L^−1^ timentin or 400–800 mg L^−1^ Amoxiclav^®^ to the culture medium.

## Figures and Tables

**Table 1:** Types and concentrations of β-lactam antibiotics used in MS culture media for in vitro callus induction and shoot organogenesis of tomato.

Culture Medium		MS2	MS3	MS4	MS5	MS6	MS7	MS8	MS9
Antibiotic	brand name	Claforan^®^, Sanofi-Aventis (France)		Timentin, PhytoTechnology Laboratories (USA)		Amoxicillin, PhytoTechnology Laboratories (USA)		Amoxiclav^®^, Lek d.d. (Slovenia)	
	generic name	Cefotaxime sodium		Ticarcillin disodium + clavulanate potassium (15:1)		Amoxicillin		Amoxicillin + clavulanate potassium	
Concentration, mg L^−1^		400	800	100	200	200	400	400	800

**Table 2:** The effects of genotype, explant type, and culture medium components on the frequency of tomato shoot organogenesis in vitro.

Culture Medium	Antibiotic and Its Concentration, mg L^−1^		Frequency of Shoot Organogenesis, %						
			cv. Moryana			cv. Rekordsmen			Mean (Culture Medium) ^3^
			Hypocotyls	Cotyledons	Mean ^2^	Hypocotyls	Cotyledons	Mean	
MS1	—		4.4 ^1^ ab	67.1 fghijk	35.8 cdef	1.5 ab	42.7 fghij	22.1 c	29.0 b
MS2	Claforan^®^	400	4.3 ab	71.6 ijk	38.0 cdef	11.9 bcde	1.2 ab	6.6 b	22.3 b
MS3	Claforan^®^	800	0 a	56.8 efghij	28.4 c	0.8 ab	0 a	0.4 a	14.4 a
MS4	Timentin	100	37.4 de	77.5 jk	57.5 g	17.6 cdef	46.6 ghij	32.1 cde	44.8 d
MS5	Timentin	200	16.8 bcd	89.2 k	53.0 efg	26.5 defgh	80.6 l	53.6 fg	53.3 de
MS6	Amoxicillin	200	30.2 cde	81.6 jk	56.0 fg	30.6 efgh	68.4 jkl	49.5 efg	52.8 de
MS7	Amoxicillin	400	10.9 bc	87.0 k	54.4 defg	6.7 abcd	68.4 ijkl	37.6 cdef	46.0 cd
MS8	Amoxiclav^®^	400	10.9 bc	70.3 hijk	46.1 cdefg	77.8 kl	100 mn	88.9 i	67.5 f
MS9	Amoxiclav^®^	800	6.7 ab	67.1 ghijk	36.9 cdef	55.7 hijkl	100 n	77.9 hi	57.4 ef
Mean (explant) ^4^	—		13.5 a	74.2 b	—	25.5 a	56.4 b	—	—
Mean (genotype) ^5^	—		43.9 a			40.9 a			—

Means followed by the same letter are not significantly different at α = 0.05 according to the Duncan’s multiple range test. Data were arcsin $\sqrt{X}$ transformed prior to statistical analysis. ^1^ Influence of culture medium component and explant type on the shoot organogenesis frequency. ^2^ Influence of culture medium component and genotype on the shoot organogenesis frequency. ^3^ Influence of culture medium component on the shoot organogenesis frequency. ^4^ Influence of explant type on the shoot organogenesis frequency. ^5^ Influence of genotype on the shoot organogenesis frequency.

**Table 3:** The effects of genotype, explant type, and culture medium components on the average number of tomato shoots produced per explant in vitro.

Culture Medium	Antibiotic and Its Concentration, mg L^−1^		Average Number of Shoots per Explant						
			cv. Moryana			cv. Rekordsmen			Mean (Culture Medium) ^3^
			Hypocotyls	Cotyledons	Mean ^2^	Hypocotyls	Cotyledons	Mean	
MS1	—		1.9 ^1^ bc	2.0 bcde	2.0 bcd	1.3 ab	2.3 bcde	1.8 abc	1.9 ab
MS2	Claforan^®^	400	1.6 ab	3.7 ghi	2.7 bcdefg	2.6 bcde	1.5 abcd	2.0 bcde	2.4 bc
MS3	Claforan^®^	800	1.0 a	2.3 bcdef	1.7 ab	1.3 ab	1.0 a	1.1 a	1.4 a
MS4	Timentin	100	3.0 cdefghi	5.6 j	4.3 jkl	2.0 abcde	2.3 bcde	2.2 bcdef	3.2 cd
MS5	Timentin	200	2.9 cdefghi	4.3 ij	3.6 gh6ijk	2.9 cde	5.3 fgh	4.1 ijk	3.8 d
MS6	Amoxicillin	200	2.6 bcdefg	3.5 fghi	3.0 defghi	3.0 de	3.5 efg	3.3 fghij	3.1 cd
MS7	Amoxicillin	400	2.2 bcde	3.2 efghi	2.7 cdefg	2.0 abcde	9.2 i	5.6 l	4.2 d
MS8	Amoxiclav^®^	400	2.0 bcd	3.1 defghi	2.6 bcdefg	3.2 e	6.3 h	4.8 kl	3.7 d
MS9	Amoxiclav^®^	800	2.3bcdef	4.0 hi	3.1 efghij	2.7 bcde	5.3 gh	4.0 hijk	3.6 d
Mean (explant) ^4^	—		2.0 a	3.5 b	—	2.0 a	4.0 b	—	—
Mean (genotype) ^5^	—		2.8 a			3.0 a			—

Means followed by the same letter are not significantly different at α = 0.05 according to the Duncan’s multiple range test. The average number of shoots per explant were $\sqrt{X+1}$ transformed prior ANOVA. ^1^ Influence of culture medium component and explant type on the number of shoots per explant. ^2^ Influence of culture medium component and genotype on the number of shoots per explant. ^3^ Influence of culture medium component on the number of shoots per explant. ^4^ Influence of explant type on the number of shoots per explant. ^5^ Influence of genotype on the number of shoots per explant.

## Data Availability

Data sharing is not applicable to this article.

## Supplementary Materials

The following are available online at https://www.mdpi.com/article/10.3390/antibiotics10060660/s1, Table S1: The results of three-way ANOVA test to evaluate the significance of culture medium components, tomato genotype and explant type on the callus formation frequency; Figure S1: Antibacterial activity of different β-lactam antibiotics on growth inhibition of *A. tumefaciens* strain AGL0 using agar disk-diffusion assay.
