# Supplementary material for: Effects of Different β-Lactam Antibiotics on Indirect Tomato (Solanum lycopersicum L.) Shoot Organogenesis and Agrobacterium tumefaciens Growth Inhibition In Vitro
Source: Antibiotics (Basel). 2021 Jun 1;10(6):660. doi: 10.3390/antibiotics10060660 (PMC8229254; doi:10.3390/antibiotics10060660)
Supplement: Supplementary file 1 [file antibiotics-10-00660-s001.zip › Figure S1.pdf]

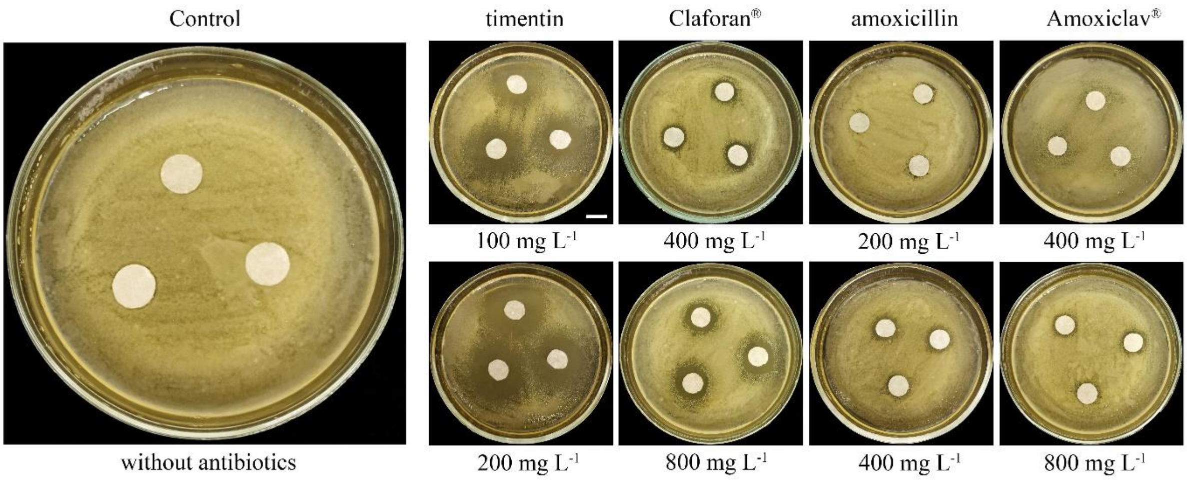

Figure S1. Antibacterial activity of different β-lactam antibiotics on growth inhibition of *A. tumefaciens* strain AGL0 using agar disk-diffusion assay.
