# Supplementary material for: Effects of Different β-Lactam Antibiotics on Indirect Tomato (Solanum lycopersicum L.) Shoot Organogenesis and Agrobacterium tumefaciens Growth Inhibition In Vitro
Source: Antibiotics (Basel). 2021 Jun 1;10(6):660. doi: 10.3390/antibiotics10060660 (PMC8229254; doi:10.3390/antibiotics10060660)
Supplement: Supplementary file 1 [file antibiotics-10-00660-s001.zip › Table S1.pdf]

Table S1. The results of three-way ANOVA test to evaluate the significance of culture medium components, tomato genotype and explant type on the callus formation frequency.

Source of variation	ss	df	ms	F_{05}	F
Total	35652.04	107	–	–	–
Variants	24319.19	35	694.83	1.53	4.41*
Factors					
A (culture medium)	8382.13	8	1047.77	2.02	6.66*
B (explant)	1789.50	1	1789.50	1.93	11.37*
C (genotype)	138.31	1	138.31	1.93	0.88
Interactions					
AB	3911.10	8	488.89	2.02	3.11*
AC	2691.08	8	336.38	2.02	2.14*
BC	785.24	1	785.24	1.93	4.99*
ABC	6621.83	8	827.73	2.02	5.26*
Error	11332.86	72	157.40	–	–

Abbreviations: ss – sum of squares, df –degrees of freedom, ms – mean square, F_{05} – critical F value at 5% significance level ($\alpha = 0.05$), F – F value, * – F test significant at $\alpha = 0.05$.
